# Prevalence and Associated Positive Psychological Variables of Depression and Anxiety among Chinese Cervical Cancer Patients: A Cross-Sectional Study

**DOI:** 10.1371/journal.pone.0094804

**Published:** 2014-04-10

**Authors:** Yi-Long Yang, Li Liu, Xiao-Xi Wang, Yang Wang, Lie Wang

**Affiliations:** 1 Department of Social Medicine, School of Public Health, China Medical University, Shenyang, Liaoning, PR China; 2 School of Clinical Medical and Pharmaceutical College, China Medical University, Shenyang, Liaoning, PR China; Institute of Psychiatry, United Kingdom

## Abstract

**Background:**

The prevalence of depression and anxiety and its associated factors in cervical cancer are not well evaluated in China. Meanwhile, with increasing attention given to positive psychological variables in oncology field, there is a need to conduct a study to explore the integrative effects of positive psychological variables on depression/anxiety so as to provide patients a more holistic cancer care. The aim of this study was to assess the prevalence of depression/anxiety as well as the integrative effects of hope, optimism and general self-efficacy on depression/anxiety among Chinese cervical cancer patients.

**Methods:**

A multi-centre, cross-sectional study was conducted of consecutive inpatients at the Liaoning Cancer Hospital & Institute and the Shengjing Hospital of China Medical University in Liaoning Province, northeast China. A total of 224 cervical cancer patients eligible for this study completed questionnaires on demographic and clinic variables, Hospital Anxiety and Depression Scale, Herth Hope Index, Life Orientation Scale-Revised, and General Self-Efficacy Scale during February and August 2013.

**Results:**

The prevalence of depression and anxiety was 52.2% and 65.6% in cervical cancer patients. The anxiety score was significantly higher in patients at the period of 4–6 months after diagnose and at cancer stage II. Hierarchical regression analyses indicated that hope, optimism and general self-efficacy as a whole accounted for 31.3% variance of depression and 35.6% variance of anxiety. Under standardized estimate (β) sequence, hope, optimism and general self-efficacy significantly associated with depression, respectively; hope and optimism were also significant individual predictors of anxiety.

**Conclusions:**

The high prevalence of depression and anxiety among cervical cancer patients should receive more attention in Chinese medical settings. More importantly, efforts to develop the integrated psychosocial interventions are effective and necessary to alleviate depression/anxiety in cervical cancer patients by synthesizing and integrating the individual protective effects of hope, optimism and general self-efficacy.

## Introduction

Cancer is a serious and potentially life-threatening illness, which has negative effects on physical and psychological well-being of patients. The diagnosis and treatment of cancer considered as a major life stress can cause or aggravate the related psychological distress [1], [2]. Some systemic reviews have indicated that depression and anxiety were two of the common psychological distress in cancer patients [1]–[4]. Our previous meta-analysis also found that the prevalence of depression (54.90% vs. 17.50%) and anxiety (49.69% vs. 18.37%) were significantly higher in Chinese adults with cancer compared with those without [5]. More importantly, the unrecognized and untreated depression and anxiety could lead to difficulty with symptom control, poor compliance with treatment, prolonged recovery times and impaired quality of life [3], [5], [6].

Besides the general impacts of cancer diagnosis and treatment, cervical cancer patients have some noticeable differences of psychological and social states. Cervical cancer was the first and second most common cancer among women in China and around the world [7]–[9]. Because of the cancer site and treatment of organs accomplishing reproductive and hormonal functions, cervical cancer patients tended to have problems including their self-identity and self-image, female fertility, and changes in sexual function [10], [11]. Besides, cervical cancer is mainly caused by sexually transmitted infection (STI) of human papillomavirus (HPV), therefore, cervical cancer patients could be associated with negative labels by general public, including promiscuous, not willing to have protected sex, and even unfaithfulness, and patients themselves also expressed shame, self-blame and the fear of social exclusion [12]. These unique characteristics of cervical cancer might aggravate psychological distresses among patients, in addition to the general distress that follows cancer diagnose and treatment. Among gynecological cancer, cervical cancer patients reported the worst scores in terms of emotional distress and quality of life [10], [13]. A longitudinal study showed that the prevalence of depression and anxiety was 7.4%-11.4% and 53.4%–62.9% among cervical cancer patients at baseline [14].

Because of the high prevalence and the negative impacts of depression/anxiety, a lot of studies mainly explored the variables influencing depression and anxiety among cancer patients. In addition to the impacts of demographic and clinical variables on depression and anxiety [14]–[16], positive psychological variables started to receive increasing attention in oncology field over the last 20 years. According to the literature review, we found that hope, optimism and general self-efficacy were key research topics in this field.

Hope is considered as a important and positive factor in the lives of cancer patients, including helping to adjust to cancer and reducing psychological distress [17], [18]. Defined by Dufault and Martocchio, hope was a multidimensional dynamic resource characterized by a confident yet uncertain expectation of achieving a future good which is realistically possible and personally significant [19]. This classic definition has assisted to describe hope in cancer patients [20]. Based upon the six dimensions of hope conceptualized by Dufault and Martocchio [19], Herth further developed a hope model including three dimensions: temporality and future (the perception that a positive, desired goal is realistically probable in the future); positive readiness and expectancy (a feeling of confidence with initiation of plans to achieve the desired goal); interconnectedness (the recognition of the interdependence between self and others) [21]. This model and its relevant scale were commonly used to measure and analyze hope in cancer patients [17], [18], [20], [21].

Optimism also appears to be an important prospective predictor of psychological distress among cancer patients [22], [23]. Optimism was defined as a relatively stable generalized tendency to expect that good rather than bad things will happen, and the general positive outcome expectation will cause or enhance the continuous efforts in achieving the desired positive goal [22], [24]. Therefore, optimism could help individuals to better adjust to negative life events and serious illnesses, including cancer [25]. A recent review indicated that, compared with pessimists, optimists mainly use adaptive and positive coping styles to deal with cancer, including accepting the reality, placing a positive light, and humor [26]. It is noteworthy that the desired goal expected by cancer patients should be realistic and achievable, otherwise optimism may be useless or even bring patients bad influences [26], [27].

General self-efficacy, as a derivative construct of self-efficacy, referred to a relatively stable belief of personal competence to deal effectively with a variety of stressful situations [28]. Different from the original definition of self-efficacy considered as being situation-specific [29], general self-efficacy mainly reflected a generalized positive belief in one's ability to achieve goals or deal with the challenges across various situations [28]. General self-efficacy was considered as an important psychological resource that has been examined in relation to patient's adjustment to cancer [30]. Therefore, the construct of general self-efficacy has been more and more applied and evaluated in different types of cancer patients [30]–[32].

To date studies in the oncology field have mainly explored the impact of one of the three variables (hope, optimism and general self-efficacy) on depression and anxiety among cancer patients [17], [23], [31]. Although these three constructs were defined in different ways and might influence depression and anxiety through different mechanisms, they had at least three points in common. First, to some extent, they could all refer to the internally positive psychological constructs (or called psychological resource) of individual; second, they all could be considered as a positive coping style (or they co-occurred with more adaptive and positive coping strategies) with an illness experience including cancer [18], [23], [26], [30], [33]; third, they all could be recognized as a positive personality, or they could be supposed to be relatively stable (or called trait like) constructs [18], [22], [23], [26], [28], [31].

While depression and anxiety are recognized problems among Chinese cancer patients [6] and cervical cancer patients may have the higher level of depression/anxiety, few studies used a relatively large sample (n>200) and multi-centre sampling to assess depression/anxiety and the associated factors in Chinese cervical cancer patients. Likewise, positive psychological constructs are getting attention in oncology field, but there are very few studies exploring the integrative effects of the associated positive psychological variables on depression and anxiety. The aim of the present study was to assess the depression/anxiety among cervical cancer patients and to clarify the associated factors (demographic and clinical variables). More importantly, we aimed to confirm the integrative effects of hope, optimism and general self-efficacy on depression and anxiety after adjusting for the demographic and clinical variables.

## Methods

### Ethics statement

The Committee on Human Experimentation of China Medical University reviewed and provided the ethics approval for this study, and the study procedures were in accordance with the ethical standards. All the patients gave their written informed consent to participate after being orally informed about the study protocol, and they were totally voluntary and anonymous. We protected the privacy of patients in processing personal data and maintained confidentiality of individual records and accounts. The participation in this study did not affect the future free health examination and treatment which is standard in China.

### Study design and study sample

During February and August 2013, a multi-centre, cross-sectional study was conducted of consecutive inpatients with cervical cancer. The study took place in the Department of Gynecology at Liaoning Cancer Hospital & Institute and the Department of Obstetrics and Gynecology at Shengjing Hospital of China Medical University, which are the two important providers of cancer services to a geographically defined area of 8.2 million people in the south of northeastern China. The requirements for participation in this study were that patients (1) were at least 18 years old, (2) had histologically proven cervical cancer, (3) were aware of their own cancer diagnosis, (4) were able to communicate in Chinese language well enough to answer the questionnaires, (5) had clear consciousness and cognition (be able to accurately answer questions on persons, place, and time within 30 seconds). Exclusion criteria were the following: (1) patients had a history of psychiatric problems (e.g., depression, anxiety, and other psychiatric disorders) before cancer diagnose, (2) patients had intellectual impairments, (3) patients had other active cancers. All eligible patients were invited to participate in the study by their treating oncologists or attending physicians. Researchers confirmed that patients were well-informed about the purpose and steps in the study. After obtaining patient written consent, clinical data were collected from the medical record, and a structured questionnaire was distributed to patients. Initially, a total of 253 patients were enrolled. Four patients refused to participate, and four patients had other active cancers (ovarian cancer, lung cancer, vaginal cancer, bladder cancer). Of 245 eligible patients for this study, 21 excluded from analysis (>30% missing data). Finally, we received effective responses from 224 cervical cancer patients with effective response rate 91.4%. These patients became our subjects.

### Measurements of depression and anxiety

The Hospital Anxiety and Depression Scale (HADS) consisted of 14-items, which was one of the most commonly used instruments worldwide for assessing anxiety and depression in clinical patients with physical problems [34], and the Chinese version of HADS had good reliability and validity among patients with cancer and other somatic diseases [22], [35]. The HADS consisted of two subscales, anxiety and depression, with seven items each. Participants rated on a 4-point Likert scale (0 = not at all and 3 = very much indeed). The scores of each subscale ranged from 0 to 21, with higher scores reflecting higher anxiety and depression. Participants were grouped as follows: 0–7 = normal, 8–10 = possible anxiety and depression, 11–21 = probable anxiety and depression, according to the cut-off values recommended by Zigmond and Snaith [34]. In our study, unless otherwise stated, anxiety and depression referred to patients whose scores were 8 or greater as rated on the subscales of HADS, and this cut-off was an optimal balance between sensitivity and specificity in most studies [36]. The Cronbach's α value for total 14 items was 0.792, and Cronbach's α values for anxiety and depression subscales were 0.728 and 0.615.

### Measurement of hope

The Herth Hope Index (HHI), a 12-item scale adapted version of the Herth Hope Scale (HHS) [37], was designed to assess hope in adults in clinical settings [21]. The HII delineated three factors of hope: a) temporality and future, b) positive readiness and expectancy, and c) interconnectedness [21]. Each item was rated from 1 (strongly disagree) to 4 (strongly agree) and total scores range from 12 to 48, with a higher score reflecting greater hope. The Chinese version of HHI has demonstrated the test-retest reliability, internal consistency, content validity and construct validity in cancer patients [38]. In this study, the Cronbach's α value for total 12 items was 0.880.

### Measurement of optimism

The Life Orientation Scale-Revised (LOT-R), originally developed by Scheier and Carver [24], was used to assess optimism [39]. The scale comprised 6 items (along four filler items) containing three positive-worded items for positive general life expectations and three negative-worded items for negative general life expectations. Participants rated on a 5-point Likert scale, ranging from 1 (strongly disagree) to 5 (strongly agree). The total sum score (ranging from 6 to 30) was calculated by the addition of raw scores of three positive-worded items and the inverted raw scores of three negative-worded items. This scale has been applied well to Chinese cancer patients [22], [40]. In this study, the Cronbach's α value for this scale was 0.831.

### Measurement of general self-efficacy

The General Self-Efficacy Scale (GSES) was employed to assess patients' general self-efficacy [41], and the Chinese version was validated by Zhang and Schwarzer [42]. This scale consisted of 10 items rated on a 4-point scale, ranging from 1 (not at all true) to 4 (exactly true). The total score ranged from 10 to 40 scores, and higher score indicated higher level of general self-efficacy. This scale has also been adapted to Chinese cancer patients [42], [43]. The Cronbach's α value for general self-efficacy in the current study was 0.911.

### Measurements of demographic and clinical variables

We collected the demographic variables of patients including age, marital status, educational level and income. We also surveyed the clinical variables about time (number of months) since diagnosis, cancer stage, treatment type and cancer metastasis. In our study, cancer stage was defined as the International Federation of Gynecology and Obstetrics (FIGO). FIGO stage I carcinoma is strictly confined to the cervix, stage II carcinoma invades beyond the uterus, stage III carcinoma extends to the pelvic wall and/or involves lower third of the vagina, and stage IV carcinoma extends beyond the pelvis or has involved the mucosa of the bladder or rectum [44], [45].

### Statistical analysis

The Statistical Package for the Social Sciences (SPSS, version 13.0) was used to perform the statistical analyses, with two-tailed probability value of <0.05 considered to be statistically significant. Descriptive statistics of the demographic, clinical and psychological variables were indicated with median, mean, standard deviation (SD), number (N) and percentage (%) as appropriate. For the categorical variable, groups for which the response rate was less than 5% were combined. In this study, only 8 participants (3.6%) belonged to the “Stage IV” group, so this group was combined with the “Stage III” group. Variations in depression and anxiety were examined with respect to demographics and clinical variables using independent sample t-test and one way analysis of variance (ANOVA). When one-way ANOVA was found to be significant, least-significant-difference (LSD) was done to perform multiple comparisons. Pearson's correlation was used to examine correlations among psychological variables. Hierarchical regression analysis was used to explore the effects of hope, optimism and general self-efficacy on depression and anxiety with adjustment for demographics and clinical variables. The demographics and clinical variables related to depression and anxiety in univariate analysis (P<0.05) were entered into step 1 of the hierarchical regression analysis. We provided data including R^2^, adjusted R^2^ (Adj.R^2^), R^2^-changes, F, standardization regression coefficient (β) and P value for each step in the regression model. Moreover, tolerance and variance inflation factor were used to check for multicollinearity.

## Results

### Descriptive statistics

Demographic and clinical variables of participants were shown in Table 1. The participants (N = 224) were in the age range of 22–79 (Mean±SD: 49.16±10.11). Approximately 90% of the participants were married or living with a partner, and 36.6% received middle school education. In relation to clinical variables, the mean number of months after diagnosis was 7.04 (range: 1–56). Minorities of participants (29.5%) were diagnosed at cancer stage III and stage IV, and 65.2% received combined treatment (a combination of different treatment modalities). Most participants were free of metastases (88.4%).

**Table 1:** Demographic and clinical variables of participants (N = 224).

	N	%
**Demographic variables**		
Age (years)		
≤35	23	10.3
36–45	46	20.5
46–55	105	46.9
≥56	50	22.3
Mean (SD)	49.16 (10.11)	
Median (Range)	49.57 (22–79)	
Marital status		
Married/living with a partner	201	89.7
Single/widowed/divorced	23	10.3
Educational level		
Primary school	41	18.3
Middle school	82	36.6
High school	50	22.3
Junior college or above	51	22.8
Income (yuan per month)		
≤1000	39	17.4
1001–2000	57	25.4
2001–3000	56	25.0
3001–4000	47	21.0
≥4001	25	11.2
**Clinical variables**		
Time since diagnosis (months)		
≤3	117	52.2
4–6	41	18.3
7–12	24	10.7
>12	42	18.8
Mean (SD)	7.04 (9.23)	
Median(Range)	3.00 (1–56)	
Cancer stage		
I	63	28.1
II	95	42.4
III+IV	66	29.5
Treatment type		
No treatment	15	6.7
Radiation therapy	28	12.5
Chemotherapy	17	7.6
Surgery	18	8.0
Combined treatment	146	65.2
Metastasis		
No	198	88.4
Yes	26	11.6

SD  =  Standard deviation.

Table 2 provided the levels of hope, optimism, general self-efficacy, depression and anxiety. Based on the cut-off values recommended by Zigmond and Snaith [34], the prevalence of depression and anxiety in cervical cancer patients was 52.2% (possible cases: 32.6%; probable cases: 19.6%) and 65.6% (possible cases: 24.1%; probable cases: 41.5%), and the prevalence of co-morbidity for both anxiety and depression was 45.5% (N = 102). Mean scores of anxiety and depression fell above the 7 point (HADS-Anxiety: Mean±SD = 9.17±3.95; HADS-Depression: Mean±SD = 7.17±3.74). The mean values were 34.62±6.57 for hope, 19.86±3.03 for optimism and 24.70±6.51 for general self-efficacy.

**Table 2:** Descriptive statistics for depression, anxiety, hope, optimism, and general self-efficacy (N = 224).

Variables	Mean	Standard deviation	Range	N (%)
**HADS-Depression**	7.17	3.74	0–19	
** Scores = 8–10**				73 (32.6)
** Scores≥11**				44 (19.6)
**HADS-Anxiety**	9.17	3.95	0–17	
** Scores = 8–10**				54 (24.1)
** Scores≥11**				93 (41.5)
**HADS**	16.33	6.87	0–30	
**HHI**	34.62	6.57	21–48	
**LOT-R**	19.86	3.03	13–30	
**GSES**	24.70	6.51	11–40	

HADS  =  Hospital Anxiety and Depression Scale; HHI  =  Herth Hope Index; LOT-R  =  Life Orientation Scale-Revised; GSES  =  General Self-Efficacy Scale.

### Effects of demographic and clinical variables on depression and anxiety

Independent sample t-test and one-way ANOVA failed to indicate the statistically significant relationships between depression, anxiety and demographic variables (P>0.05), and the statistically significant associations between depression and clinical variables were also not observed (In Table 3). However, as shown in Table 3, results indicated that participants whose time since diagnosis was in the range of 4 to 6 months had a higher level of anxiety (Mean±SD: 10.63±3.68) than the participants whose time since diagnosis was within 3 months (Mean±SD: 8.59±3.97). Results also revealed that participants at cancer stage II had higher scores of anxiety (Mean±SD: 9.85±3.79) than them at cancer stage I (Mean±SD: 8.08±4.32).

**Table 3:** Mean scores of anxiety and depression according to clinical variables.

Clinical variables	HADS-Anxiety	F/t value	P value	HADS-Depression		F/t value	P value
**Time since diagnosis (months)**		2.798	0.041		1.501		0.215
≤3	8.59±3.97^a^			6.68±3.66			
4–6	10.63±3.68^b^			7.90±3.68			
7–12	9.21±3.92			7.67±3.53			
>12	9.31±3.95			7.55±4.04			
**Cancer stage**		3.915	0.021			1.465	0.233
I	8.08±4.32^a^			6.51±3.94			
II	9.85±3.79^b^			7.33±3.49			
III+IV	9.21±3.64			7.58±3.86			
**Treatment type**		1.708	0.149			0.164	0.956
No treatment	11.67±2.29			7.67±3.44			
Radiation therapy	9.25±3.65			6.89±3.54			
Chemotherapy	9.06±3.65			7.35±3.35			
Surgery	9.28±4.30			6.78±3.42			
Combined treatment	8.89±4.09			7.20±3.92			
**Metastasis**		−0.353	0.724				
No	9.13±3.79			7.14±3.76		−0.367	0.714
Yes	9.42±3.89			7.42±3.66			

HADS  =  Hospital Anxiety and Depression Scale.

a,b Calculated by least-significant-difference (LSD), mean scores for anxiety with unequal superscripts differ significantly at the P<0.05 level.

### Correlation between hope, optimism, general self-efficacy, depression and anxiety

Pearson's correlation coefficients were calculated between depression, anxiety, hope, optimism and general self-efficacy. As shown in Table 4, depression was negatively associated with the three positive psychological variables (hope: r = −0.507, P<0.01; optimism: r = −0.420, P<0.01; general self-efficacy: r = −0.397, P<0.01). A similar pattern was also observed between anxiety and the three variables (hope: r = −0.587, P<0.01; optimism: r = −0.471, P<0.01; general self-efficacy: r = −0.293, P<0.01).

**Table 4:** Correlation among depression, anxiety, hope, optimism, and general self-efficacy.

Variables	1	2	3	4	5
**1. HADS-Depression**	1	0.596**	−0.507**	−0.420**	−0.397**
**2. HADS-Anxiety**		1	−0.587**	−0.471**	−0.293**
**3. HHI**			1	0.580**	0.445**
**4. LOT-R**				1	0.266**
**5. GSES**					1

**p<0.01.

HADS  =  Hospital Anxiety and Depression Scale; HHI  =  Herth Hope Index; LOT-R  =  Life Orientation Scale-Revised; GSES  =  General Self-Efficacy Scale.

### Hierarchical regression analyses

Two hierarchical regression analyses were conducted to explore the integrative effects of hope, optimism and general self-efficacy on depression and anxiety after adjusting for demographic and clinical variables. Because univariate analysis failed to identify any significant associations between demographic and clinical variables and depression, age considered as a control variable was entered into step 1 when depression was the dependent variable. Because of the significant effects of time since diagnosis and cancer stage on anxiety, age, time since diagnosis and cancer stage (cancer stage was represented as dummy variables), as control variables, were entered into step 1 when anxiety was dependent variable.

As shown in Table 5, hope, optimism and general self-efficacy together accounted for an additional 31.3% variance to the prediction of depression in step 2. The test of R^2^-change was significant (F Change (3, 219) = 33.523, P = 0.000), suggesting that hope, optimism and general self-efficacy, as a whole, were the significant predictors of depression. Hope (β = −0.299, P = 0.000), optimism (β = −0.188, P = 0.007) and general self-efficacy (β = −0.215, P = 0.001) also represented significantly individual predictive values. Tolerance (range: 0.564–0.973) and variance inflation (range: 1.028–1.774) did not indicate a multicollinearity problem.

**Table 5:** Hierarchical regression analysis for exploring the effects of hope, optimism and general self-efficacy on depression.

Variables	HADS-Depression (β)	
	Step 1	Step 2
**Control variables**		
Age	0.064	0.026
**Positive psychological variables**		
Hope		−0.299***
Optimism		−0.188**
General self-efficacy		−0.215***
**F**	0.921	25.474***
**R^2^**	0.004	0.318
**Adj.R^2^**	0.000	0.305
**R^2^-changes**	0.004	0.313

**p<0.01, ***p<0.001.

HADS  =  Hospital Anxiety and Depression Scale; Adj.R^2^  =  adjusted R^2^.

Hope, optimism and general self-efficacy together accounted for an additional 35.6% variance to the prediction of anxiety in step 2 (see Table 6). The test of R^2^-change was significant (F Change (3, 216) = 42.362, P = 0.000). Hope (β = −0.451, P = 0.000) and optimism (β = −0.185, P = 0.005) were individual predictive variables to anxiety, but general self-efficacy (β = −0.057, P = 0.349) was not significantly associated with anxiety. Tolerance (range: 0.558–0.954) and variance inflation (range: 1.048–1.791) did not indicate a multicollinearity problem.

**Table 6:** Hierarchical regression analysis for exploring the effects of hope, optimism and general self-efficacy on anxiety.

Variables	HADS-Anxiety (β)	
	Step 1	Step 2
**Control variables**		
Age	−0.052	−0.093
Time since diagnosis (months)	0.066	−0.010
Cancer stage 1	0.246**	0.181**
Cancer stage 2	0.150	0.104
**Positive psychological variables**		
Hope		−0.451***
Optimism		−0.185**
General self-efficacy		−0.057
**F**	2.286	20.202***
**R^2^**	0.040	0.396
**Adj.R^2^**	0.023	0.376
**R^2^-changes**	0.040	0.356

**p<0.01, ***p<0.001.

HADS  =  Hospital Anxiety and Depression Scale; Adj.R^2^  =  adjusted R^2^.

Cancer stage 1: Stage II vs. Stage I.

Cancer stage 2: Stage III+IV vs. Stage I.

## Discussion

To date, a majority of studies indicated that diagnosis and treatment of cancer [1], [2], physical state (e.g., fatigue, pain, and functional impairment) [46], [47] and other negative events [48] were primarily potential causes of depression and anxiety. Compared with the negative and inevitable factors mentioned above, positive psychological variables (hope, optimism and self-efficacy) may be secondary to depression and anxiety of cancer patients. Although some studies explored the impacts of positive psychological variables on depression and anxiety among cancer patients [17], [23], [31], the possibility that reduced hope, lower optimism and reduced self-efficacy may be also the potential causes of depression and anxiety did not receive much consideration. Therefore, in addition to discussing the prevalence and associated clinic variables of depression/anxiety among cervical cancer patients, we also mainly explored the effects of hope, optimism and self-efficacy on depression and anxiety.

### Prevalence of depression and anxiety in cervical cancer patients

The prevalence of depression and anxiety in cervical cancer patients was 52.2% (possible cases: 32.6%; probable cases: 19.6%) and 65.6% (possible cases: 24.1%; probable cases: 41.5%), and the prevalence of co-morbidity was 45.5% in our study, indicating that depression and anxiety coexisted in Chinese cervical cancer patients, similar to this situation in China and foreign countries [5], [49]. This phenomenon should be noticed because co-morbid anxiety and depressive disorders tended to have severe symptoms, poorer outcomes and greater use of healthcare resources than those with a single disorder [50]. Meanwhile, the results were similar to the meta-analysis assessing the depression (54.90%, range: 20%–89%) and anxiety (49.69%, range: 20%–89.13%) in Chinese adults with cancer [5].

We also compared our results with other reviews and empirical studies of depression and anxiety in cancer patients. These reviews were the following: (1) Massie indicated the prevalence of depression (12%–23%) of gynecologic cancer patients [2]; (2) Based on 13 literatures, Thompson showed the prevalence of depression (4%–44%) and anxiety (4%–41%) among gynecologic cancer patients [51]. The empirical studies using the same instruments (HADS) were as follows: (1) Hopwood reported that the prevalence of depression and anxiety were 33% (possible cases: 16%; probable cases: 17%) and 34% (possible cases: 17%; probable cases: 18%) in 987 lung cancer patients [46]; (2) Albrecht reported that depression occurred in 14% (possible cases: 6.3%; probable cases: 7.4%) in 175 melanoma patients [52]; (3) Based on the cut-off of 8, Kim reported that the prevalence of depression, anxiety and co-morbidity was 34.6%, 39.5% and 23.4% in 828 cervical cancer survivors in Korea [53]. By contrast, the prevalence of depression and anxiety in our study was at a high level, and there might be four reasons for the different prevalence. First explanation might be that our results may be overestimated due to the relatively small sample and high data fluctuation. Second, these studies (reviews and empirical studies) were mainly from developed countries which may have lower prevalence of mental health problems as compared to developing countries like China [54]. Third, many included literatures of these reviews used standardized clinical diagnosis, and the prevalence of depression and anxiety could be overestimated by self-report compared with clinical diagnoses [5]. Last explanation might be the influence of Chinese culture background. The continuity of ethnicity is one of the key points in Chinese traditional concepts, but cervical cancer, to some extent, can directly affect female fertility. Chinese society is also considered as sexually conservative, and Chinese culture places great emphasis on female reputation. Because of cervical cancer mainly caused by STI of HPV, patients could be associated with negative labels including promiscuous, unprotected sex, and even unfaithfulness [12]. These might aggravate depression and anxiety in Chinese cervical cancer patients compared with the different types of cancer patients from other countries.

### Effects of time after diagnosis and cancer stage on anxiety

In our study, compared with 3 months after diagnosis, anxiety tended to be higher in patients at the period of 4–6 months after diagnosis. A study found no significant association between anxiety and time after diagnosis among Japanese gynecologic cancer patients, indicating that anxiety might do not change over time [55]. However, our findings were supported by a recently longitudinal study. The longitudinal study indicated a significant rebound of anxiety at the period of 4–6 months [56]. Although the cross-sectional design was used, we could put forward a possible explanation that patients were in the process of emotional recovery at the first 100 days after diagnosis [57], but the anxiety of those patients in the period of 4–6 months after diagnose could reach a plateau again.

We also found that patients in stage II had the higher score of anxiety than them in stage I. After treatment, the 5-year survival rate for cervical cancer patients in stage I was 80% to 90%, and those patients might avoid the side-effect of radiotherapy and chemotherapy (early stages are mainly treated by surgery) [58]. So it was unsurprising that patients in stage I had the lowest score of anxiety. Interestingly, patients in stage II had higher score of anxiety than patients in advanced stages (III+IV) although the difference was not significant. There might be two main reasons for the phenomenon. First, advanced cancer patients could suffer greater physical and psychological distress, but because of this reason, they might get greater support and care from society, friends and family. Contrary to general expectation, anxiety of advanced cancer patients was often not caused by the fear of death but by the issues of the isolation and abandonment [59]. Second, doctor might not inform the patients about the true condition, but rather directly informed the family of patients when patients were suffering advanced cancer. A study indicated that anxiety and depression were higher in those who knew their cancer diagnosis than those who did not among gastrointestinal cancer patients [60].

### Hope, optimism and general self-efficacy predicting depression and anxiety

The important result of the current study was that hope, optimism and general self-efficacy accounted for a moderate proportion of variance in depression (31.3%) and anxiety (35.6%). More importantly, the three positive psychological variables as a whole had the stronger predictive values than each of them (data not shown), indicating that the integrative measure of hope, optimism and general self-efficacy may be more realistic and effective than using the individual construct to predict depression/anxiety. A similar study conducted by Rajandram also found the significant effects of hope and optimism on depression and anxiety in oral cavity cancer patients [22], but our study had three strengths over Rajandram's study. First, we explored the integrative effects of hope, optimism and general self-efficacy, as a positive psychological set, on depression/anxiety. Second, the sample size in our study was larger than that in Rajandram's study (N = 50). Third, HHI was commonly used instrument to assess hope in cancer patients [18], [21], but Adult Hope Scale (AHS) used in Rajandram's study mainly assessed hope in healthy population and occupational population.

Depression is a state of low mood and aversion to activity, and cancer patients with depression may present with worthlessness, hopelessness, lowered self esteem or suicidal preoccupation [5]. In this study, hope, optimism and general self-efficacy showed significantly independent effects on depression when they entered into regression together, indicating that hope, optimism and general self-efficacy as a whole might alleviate depression by enabling patients to establish positive, realistic and desired goals, have confidence to achieve the given goals and recognize the interdependent influence of others (hope) [17], [21], to expect positive health-related outcomes in the future and maintain the efforts to attain the realistic and achievable outcomes (optimism) [24], [26], to mobilize the resources required to positively manage or combat the physical and psychological challenges of cancer (general self-efficacy) [28]–[31]. These findings leaded us to believe that both positive outlooks regarding one's own goal, life and family/friends (hope), positive expectancies regarding favorable outcomes in one's own future (optimism), and positive beliefs regarding one's own ability to deal with the challenges (general self-efficacy) were important to successfully alleviate and even overcome depression in cervical cancer patients.

Anxiety is a normal reaction to cancer, but the untreated and prolonged anxiety may aggravate physical symptoms (pain and fatigue), interfere with the patients' quality of life and increase the side-effects during cancer treatment [4]. In contrast to the findings reported by Rajandram et al. [22], both hope and optimism were found to be significant individual predictors of anxiety in our study. However, general self-efficacy was not significantly correlated with anxiety. There might be two reasons for the result. First, unlike the definition of depression, anxiety is an unpleasant state of apprehending over what's about to happen and what could happen in the future [6]. General self-efficacy, which is a belief that an individual has the capability to deal with the present difficulties or challenges [31], might contribute little to solving or alleviating the anxiety and the fear about something that have not yet happened. A study also found that a self-efficacy promoting program could increase cancer patients' self-efficacy, but did not decrease anxiety [61]. Second, hope and optimism could be a kind of constructs with a future orientation, or they represent positive expectancies and attitudes about future [21], [26]. Therefore, only hope and optimism had unique and independent effects on anxiety.

### Implication

Several importantly theoretical and practical implications emerged from the findings of this study. In theory, this study provided the preliminary possibility of building a higher-order, core-positive psychological construct to combat depression and anxiety in cervical cancer patients by synthesizing and integrating the individual construct of hope, optimism and general self-efficacy. In practice, first, the high prevalence of depression and anxiety in cervical cancer patients should receive sufficient attention in Chinese medical settings; second, it was important for oncologists and physicians to pay more attention to detecting and treating anxiety of cancer patients at the period of 4–6 months after diagnose and at stage II; last and most importantly, a whole new perspective would be provided for researchers on the use of a integrated model to improve internal positive resources and alleviate depression/anxiety in cancer patients by synthesizing and integrating the individual protective effects of hope, optimism and general self-efficacy. Some studies have provided the concrete steps and advices to implement psychosocial interventions to enhance hope and optimism in cancer patients [17], [62]–[64]. Based on the Hope Process Framework, Herth further developed a hope promoting intervention consisting of four components, including 1) searching for hope, which was aimed at encouraging patients to express their fears and hopes, and assisting them to identify areas of hope in their lives, 2) connecting with others, which was aimed at helping patients to establish a sense of sustained connectedness with family/friends, 3) expanding the boundaries, which was to help patients to reflect on the meaning and purpose of life, and to identify sources of strength, 4) building the hopeful veneer, which was focused on the positive role of interaction with nature, positive memories and use of lightheartedness as strategies to engender hope [17]. Optimism can be improved by implementing the intervention that focus on active and accommodative coping strategies (like acceptance, positive refraining, and humor) and less use of denial and behavioral disengagement coping methods [62]. Especially for the advanced cancer about which little can be done, psychosocial interventions may focus more on the accommodative side of optimism than active striving side [62]. Besides that, the psychosocial interventions should improve optimism by considering the patients' situation-specific or treatment-specific expectations [63], [64]. However, there were few studies exploring the related interventions to promote general self-efficacy in cancer patients. Further studies should be conducted to prove whether the integrated psychosocial interventions based our findings and other studies are effective in cervical cancer patients as well as any other fields of oncology.

### Limitation

The present study had several limitations. First, we used a convenience sample, which limited the generalizability of the findings to other cervical cancer patients. Second, the interpretation of the results should be made with caution because we did not include a control group. If comparable control groups are involved, the level of depression and anxiety in cancer patients can be reliably and accurately determined. Third, depression and anxiety were not measured by psychiatrists or physicians according to the clinical diagnosis like ICD-10 (The International Statistical Classification of Diseases and Related Health Problems 10th Revision), CCMD (Chinese Classification of Mental Disorders) or HRSD/HRSA (Hamilton Rating Scale for Depression and Hamilton Rating Scale for Anxiety). Depression and anxiety measured by the self-report of HADS mainly referred to the depressive symptom and anxiety symptom in our study. Fourth, further studies need to be conducted to examine whether the results of the present study are suitable to the different cultural context. Fifth, data were mainly obtained using self-administered questions, so there was a possibility of recall and reporting bias. Finally, this study was based on cross-sectional design, which prevented the development of causal relationship between positive psychological variables and anxiety/depression. Further longitudinal studies are needed to validate the current findings.

## Conclusions

This study indicated the high prevalence of depression (52.2%) and anxiety (65.6%) in cervical cancer patients. Patients at the period of 4–6 months after diagnose and at stage II were associated with a higher score of anxiety. More importantly, hope, optimism and general self-efficacy as a whole had significantly predictive effects on anxiety and depression of cervical cancer patients. The findings support that depression and anxiety among cervical cancer patients should receive more attention in Chinese medical settings. The present study also put an insight into the integrative effects of hope, optimism and general self-efficacy in relation to anxiety and depression. This study provides a whole new perspective for future research building a higher-order, core-positive psychological construct by synthesizing and integrating the individual construct of hope, optimism and general self-efficacy in cancer patients so as to advocate the role of the integrated psychosocial interventions based on our findings in oncology field.
